# Impact of reduced vancomycin susceptibility on the therapeutic outcome of MRSA bloodstream infections

**DOI:** 10.1186/1476-0711-6-13

**Published:** 2007-10-30

**Authors:** Hui-min Neoh, Satoshi Hori, Mitsutaka Komatsu, Toyoko Oguri, Fumihiko Takeuchi, Longzhu Cui, Keiichi Hiramatsu

**Affiliations:** 1 Department of Bacteriology, Faculty of Medicine, Juntendo University, Hongo 2-1-1, Bunkyo-ku, Tokyo, 113-8421 Japan; 2 Infection Control Science, Faculty of Medicine, Juntendo University, Hongo 2-1-1, Bunkyo-ku, Tokyo, 113-8421 Japan; 3 Paediatrics, Faculty of Medicine, Juntendo University, Hongo 2-1-1, Bunkyo-ku, Tokyo, 113-8421 Japan; 4 Clinical Laboratory of Juntendo Hospital, Faculty of Medicine, Juntendo University, Hongo 2-1-1, Bunkyo-ku, Tokyo, 113-8421 Japan

## Abstract

**Background:**

The aim of this study was to determine whether clinical outcome of patients with methicillin-resistant *Staphylococcus aureus *(MRSA) bacteraemia was correlated with vancomycin susceptibility of the corresponding strains.

**Methods:**

A retrospective study on MRSA bacteraemia was performed at a teaching hospital between January 1998 and October 2005 by linking vancomycin susceptibility profiles of patients' isolates with hospitalization data.

**Results:**

A total of 20 out of 209 MRSA bacteraemia patients were treated with vancomycin for at least 5 days with adequate trough levels, and fulfilled the study's inclusion and exclusion criteria. Twenty-two *S. aureus *isolates from these patients' blood cultures were identified as MRSA, including two *hetero*-VISA from separate patients and two VISA with vancomycin MIC of 4 mg/L from one patient. Between patients who showed 'good' vancomycin response and patients who did not, there was a significant difference (p < 0.01) in their corresponding MRSAs' vancomycin susceptibility expressed by 'area under curve' (AUC) of population analysis. Significant correlations were found between AUC and initial vancomycin therapeutic response parameters of 'days till afebrile' (*r *= 0.828, p < 0.01) and 'days till CRP ≦ 30% of maximum' (*r *= 0.627, p < 0.01)

**Conclusion:**

Our study results caution healthcare personnel that early consideration should be given to cases with a poor vancomycin treatment response that could signify the involvement of MRSA with reduced susceptibility to vancomycin.

## 1. Introduction

Vancomycin has been the agent of choice for methicillin-resistant *Staphylococcus aureus *(MRSA) infections as it provided efficacious and promising therapy [1]. Nevertheless, with the emergence of *S. aureus *strains having intermediate resistance towards vancomycin (vancomycin-intermediate *S. aureus *[VISA]), treatment options for patients infected with these strains have become limited [2]. Hetero-VISA strains are also being reported more frequently worldwide [3]. These strains are interpreted as 'susceptible' to vancomycin using conventional MIC determination tests, but contain a sub-population of cells which can grow in the presence of > 2 mg/L vancomycin [3]. Clinical significance of hetero-VISA is still controversial [1,2]. Therefore, it is important to elucidate whether vancomycin susceptibility of clinical MRSA strains is correlated with the corresponding patients' clinical outcome. We report here the results of a retrospective study that was carried out to investigate the existence of the above correlation.

## 2. Methods

### 2.1 Setting

A retrospective study on MRSA bloodstream infections at Juntendo University Hospital, Tokyo, Japan, between January 1998 and October 2005 was carried out. The inclusion criterion of the study was defined as febrile patients (body temperature > 37°C) with at least one MRSA positive blood culture, and had been treated with a monotherapy of vancomycin for at least 5 days. In addition, serum vancomycin trough levels should have been maintained above 10 mg/L [4]. Patients who had catheter-associated MRSA bloodstream infections (CABSI) were also included in the study; however, they were excluded if their CABSI is a catheter related bloodstream infection (CRBSI), since CRBSIs are usually self-limiting, and recovery could be achieved with the removal of contaminated catheters without antibiotic treatment. The diagnosis for CABSI and CRBSI was done using the Center for Disease Control (CDC) definition of catheter-related infections for National Nosocomial Infection Surveillance (NNIS) [5].

### 2.2 Medical record review and definitions

Medical records of patients eligible for this study were reviewed to retrieve information such as demographic factors (age and gender) and their medical history. The patients' underlying conditions were then evaluated according to the American Society of Anesthesiologists (ASA) scoring system [6]. Besides this, each case's initial therapeutic response parameters towards vancomycin were recorded. The parameters used in this study were 'days till afebrile', 'days till CRP value ≦ 30% of maximum' and 'days till WBC < 10,000'. A parameter of 'days till afebrile' was defined as the number of days required for the patient's defervescence (body temperature becoming less than 37°C) after commencement of vancomycin therapy, while 'days till CRP ≦ 30% of maximum' refers to the number of days required for the reduction of patient's CRP level to less than 30% of the maximum value during active infection, after vancomycin was administered. The number of days required for patients' white blood cell count to return to the normal range of ~10,000 cells/mm^3 ^after initiation of vancomycin therapy was defined as 'days till WBC < 10,000'. If the patient had died during treatment, the above parameters would then refer to the number of days from the day of vancomycin commencement till death for each parameter. The duration of MRSA blood culture positivity during vancomycin therapy was also noted for each patient, as well as the patient's survival at one month after the onset of MRSA bloodstream infection. Patient's response to vancomycin therapy was defined as either 'good' or 'poor' [7]. 'Good' represents cases where patients were cured, with elimination of clinical symptoms and laboratory evidence of infection; patients who were apparently cured but died due to other causes were also included in this category. 'Poor' is defined for cases with persistence or worsening of symptoms and also cases where the patient died due to the bloodstream infection itself.

### 2.3 Laboratory investigations

#### 2.3.1 Bacterial strains

All MRSA blood stream isolates used in this study were recovered from hospitalized patients in Juntendo University Hospital between January 1998 and October 2005. Only the initial isolate for each patient was used for laboratory investigations. Reference hetero-VISA strain Mu3 and VISA strain Mu50 were isolated in January and September 1996, respectively, at the same hospital [3,8]. Vancomycin-susceptible MRSA strain N315 was isolated in 1982 from the pharyngeal smear of a Japanese patient [3]. Identification of *S. aureus *was confirmed by Gram's stain, catalase test and slide latex agglutination test (STAPHYLO LA "SEIKEN", Denka Seiken Co., Ltd., Japan) [9]. Strains were stocked from time of isolation in Mueller-Hinton broth with 40% glycerol at -70°C.

#### 2.3.2 Antibiotic susceptibility testing

Vancomycin minimum inhibitory concentrations (MIC) for all isolates were determined using the agar dilution method in accordance with the Clinical and Laboratory Standards Institute (CLSI) recommendations [10]. Analysis of vancomycin-resistant subpopulation (population analysis) for all strains was carried out as described previously [3]. Log graphs of viable count versus vancomycin concentrations were plotted and area under curve (AUC) was then calculated [11].

### 2.4 Statistical analysis

Independent t-tests were used to compare the means of AUC between patients showing 'good' or 'poor' vancomycin response. Pearson's correlation tests were use to determine the correlation between MRSA vancomycin susceptibilities and corresponding patients' initial therapeutic response parameters of 'days till afebrile' and 'days till CRP ≦ 30% of maximum'. All statistical analyses were performed using SPSS 11.5J for Windows (LEAD Technologies, INC.).

## 3. Results and discussion

This study was performed to investigate the relationship between vancomycin susceptibility of MRSA and the corresponding patients' clinical outcome. The true clinical relevance of hetero-resistance or intermediate resistance to vancomycin in *S. aureus *may be obscured because of uncertainty of the patients' clinical conditions. Many published case reports did not provide sufficient details to assess the role of the infection itself, nor of the antibiotic treatment in the clinical course of individual patients. Our study was designed to include only clinically significant cases of MRSA bloodstream infection that received vancomycin monotherapy, and to determine whether vancomycin susceptibility of the causative MRSA correlated with the clinical efficacy of vancomycin in the treatment of these infections. To achieve this, a strict inclusion and exclusion criteria for selection of clinical cases was employed throughout the study (see methods). Based on the study's inclusion and exclusion criteria, a total of 20 patients, with a mean age of 54.5 years comprising 12 men and 8 women, were eligible for this study. The patients' clinical history is summarized in Table 1. While these patients had various underlying diseases, they could be mainly categorized into cancer, circulatory system disorder or diabetic patients. Patients were hospitalized for a mean duration of 135.9 ± 109.03 days, while mean duration of vancomycin treatment was 13.8 ± 5.58 days, respectively. Four patients were MRSA nasal carriers when they were admitted. Eighteen patients had insertion of catheters and this was suspected to be the cause of CABSI in 10 cases.

**Table 1:** Clinical summary of patients eligible for this study

Pt no.	Sex	Age	Underlying disease	ASA^a^	Hospitalization days	VCM Treatment days^b^	Shock	Infection source	MRSA carrier^e^
1	M	58	Extraheptic bile duct carcinoma, post operation liver failure	IV	86	9	yes	SSI^c^	no
2	F	28	Pneumonia, alchoholic hepatitis	III	92	5	yes (DIC)	CVC	no
3	F	25	Multiple sclerosis, steroids therapy	III	67	20	no	transfusion	no
4	M	66	Liver cirrhosis, renal dialysis, brain stroke	IV	129	19	yes (DIC)	unknown	no
5	M	70	Renal dialysis, brain stroke, surgery in admission	IV	85	12	yes	SSI	no
6	F	91	Diabetes, chronic heart failure	IV	252	16	no	CVC^d^	yes
7	M	22	Germ cell tumour, chemotherapy	III	301	9	no	CVC	yes
8	M	63	Diabetes, brain stroke, aspiration	IV	260	11	yes	unknown	no
9	F	57	Diabetes, SLE, surgery in admission	IV	137	23	yes	pressure sore	yes
10	M	38	Lung cancer, chemotherapy, metastasis	IV	105	19	no	pressure sore	no
11	F	73	Brain stroke, aspiration, surgery in admission	IV	43	15	no	CVC	yes
12	F	3	B cell lymphoproliferative disorder, steroids	III	50	8	no	CVC	yes
13	M	77	Diabetes, chronic renal failure, aspiration	IV	281	10	no	CVC	no
14	M	59	Renal cancer, metastasis to bone, lung	IV	111	7	no	CVC	no
15	M	79	Lung cancer, chemotherapy	III	430	17	no	cellulitis	no
16	M	57	Non-Hodgkin's lymphoma, chemotherapy	IV	21	14	no	unknown	no
17	F	67	Liver cancer, liver cirrhosis, hypertension	III	69	9	no	port	no
18	F	22	Pulmonary embolism, bone tumour transplantation	III	86	19	no	transplantation	no
19	M	72	Encephalopathy, apical OMI, aspiration pneumonia	IV	59	10	no	CVC	no
20	M	62	Heart failure, acute myocardial infarct	IV	54	24	yes	CVC	no

a) ASA (American Association of Anaesthetists) scoring to assess patients' underlying conditions: I, a completely healthy patient; II, a patient with mild systemic disease; III, a patient with severe systemic disease that is not incapacitating; IV, a patient with incapacitating disease that is a constant threat to life; V, a moribundpatient who is not expected to live 24 hours with or without surgery.

b) Duration of vancomycin treatment

c) SSI, surgical site infection

d) CVC, central venous catheter

e) MRSA nasal carrier during hospital admission

To support our study design, only blood cultures were used in the laboratory investigation, strains obtained from tips or catheters were excluded. Initial isolates of all patients had vancomycin MIC levels within the susceptible range (1–2 mg/L); while two subsequent MRSA strains from patient no.15 isolated during vancomycin therapy had both vancomycin MICs of 4 mg/L each, and were judged as VISA according to CLSI criteria, Jan. 2006 [10]. Patient no. 15 was admitted on 28th October 2004 for small cell lung carcinoma. He developed MRSA cellulites, which was treated with vancomycin from 28th November. MRSA was then isolated from his blood on 3rd December. Vancomycin treatment was given until 14th December, but the patient's condition worsened. Subsequently, two VISA strains were isolated from his blood on 21st and 25th December, and these three bloodstream isolates showed identical band patterns in pulsed field gel electrophoresis (Data not shown). Finally, this patient recovered from sepsis by a combination therapy of vancomycin with arbekacin. This patient's clinical course underscores the clinical impact of hetero-VISA, generally recognized as "vancomycin-susceptible" MRSA based on MIC data alone, that cause slow and poor vancomycin therapeutic response. It is considered that hetero-VISA initiates apparently regular MRSA infection, but soon generates VISA within the patient's body during vancomycin therapy, eventually causing therapeutic failure.

**Figure 1:**
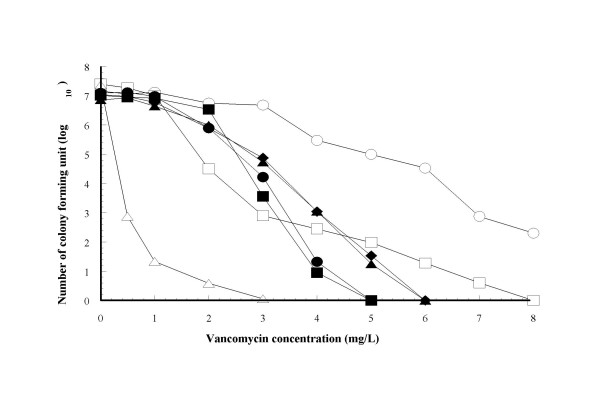
Population analysis profiles of representative strains and control strains. Symbols: open circles, strain Mu50; open squares, strain Mu3; open triangles, strain N315; closed circles, hetero-VISA 02-6; closed squares, hetero-VISA 03-10; closed triangles, VISA 04-18; closed diamonds, VISA 04-19.

Vancomycin treatment failures are not uncommon with MRSA infections despite the organism being fully susceptible (vancomycin MIC ≦ 2 mg/L) according to standard clinical laboratory testing methods [12]. Even though the clinical course of patients in this study were not entirely similar, all their initial MRSA strains had vancomycin MICs of 1–2 mg/L, which were in the 'susceptible' range. This implies the possibility, though small, that different vancomycin susceptibility levels within the 'susceptible' range may result in dissimilar clinical outcome. To discriminate in more detail the vancomycin susceptibility of these strains, we employed population analysis (PA) methods, which can provide vancomycin susceptible profile for individual cell [3]. The area under curve (AUC) for each strain's population analysis profile was calculated and presented in Table 2, along with the clinical course of each strain's corresponding patient. The AUC represents the strains' resistance level to vancomycin [11]. We found that mean AUC of strains from patients who showed 'good' vancomycin response was 10.81 ± 1.66, and those from patients who showed 'poor' vancomycin response was 16.09 ± 5.09, showing significant difference in the vancomycin susceptibility between these two patient groups' MRSA (p < 0.01). Two strains, 02-6 and 03-10 from patient no.4 and patient no.9, respectively, had high AUC values and were judged as hetero-VISA from their PA profiles [3,11]. These patients had 'poor' vancomycin treatment response and died as a result of the bloodstream infection (Table 2). Figure 1 shows PA profiles of representative strains with control strains Mu3, Mu50 and N315. This finding further emphasizes the impact of reduced MRSA vancomycin susceptibilities towards patients' clinical course in bloodstream infections.

**Table 2:** Microbiological profile of MRSA strains and clinical outcome of corresponding patients

Patient	MRSA strains	VCM MIC (mg/L)^a^	AUC^b ^(days)	DTA^c ^(days)	DTCRP^d ^(days)	DTWBC^e ^(days)	DBCP^f ^(days)	Survival at one month	VTR^g^
1	98-7	2	18.94	10	10	<10,000	n.a.	dead	Poor
2	99-7	2	12.3	10	10	> 10,000	10	dead	Poor
3	01-3	1	10.95	6	8	<10,000	n.a.	alive	Good
4	02-6	2	22.01	19	19	> 10,000	n.a.	dead	Poor
5	03-1	1	9.12	5	6	1	8	dead	Good
6	03-3	1	10.57	4	8	9	15	alive	Good
7	03-5	1	8.54	3	6	<10,000	n.a.	alive	Good
8	03-9	1	12.56	12	2	<10,000	2	dead	Good
9	03-10	2	23.11	25	12	7	13	dead	Poor
10	03-13	1	7.62	2	4	> 10,000	n.a.	dead	Good
11	03-16	1	11.56	15	12	8	n.a.	transferred	Poor
12	03-29	1	11.14	4	3	<10,000	4	alive	Good
13	04-6	1	10.88	5	9	6	16	dead	Good
14	04-13	1	9.84	4	7	<10,000	18	dead	Good
15	04-15	1	11.92	12	12	< 10,000	4	alive	Poor
16	04-17	1	12.81	14	15	<10,000	10	alive	Poor
17	05-1	1	11.39	10	8	<10,000	n.a.	discharged	Good
18	05-4	1	12.19	8	8	<10,000	11	alive	Good
19	05-10	1	13.08	6	9	no change	n.a.	alive	Good
20	05-12	1	12.59	10	6	6	9	dead	Good

a) Minimum inhibitory concentrations (MIC) for vancomycin was determined using the agar dilution method in accordance with the Clinical and Laboratory Standards Institute (CLSI) recommendations (9).

b) AUC (area under curve), measurements of polulation analysis profiles for vancomycin. Log of viable colonies were ploted against vancomycin concentration. AUC represents area covered by curve and graph axis.

c) DTA (days till afebrile), represents number of days required for the patient's defervesence (body temperature less than 37°C) after commencement of vancomycin therapy.

d) DTCRP (days till CRP ≦ 30% of maximum), refers to the number of days required for the reduction of patient's CRP level to less than 30% of the maximum value during active infection, after vancomycin was administered.

e) DTWBC (days till white blood cell < 10,000), refers to the number of days needed for patients' white blood cell level to return to the normal range of ~10,000 cell/mm^3^.

f) DBCP refers to duration of MRSA blood culture positivity during vancomycin therapy.

g) VTR (vancomycin treatment response), was defined as either 'good' or 'poor'. 'Good' repersents complete cure or elimination of clinical symptoms and elimination of laboratory evidence of infection, while 'poor' stands for persistence or worsening of symptoms or death due to MRSA bloodstream infection.

To further understand the effect of MRSA vancomycin susceptibility on the corresponding patient's clinical course, we evaluated the initial therapeutic response of each patient's infection towards vancomycin. For this evaluation, 3 parameters were used, namely 'days till afebrile', 'days till CRP ≦ 30% of maximum' and 'days till WBC < 10,000' (see methods). While the parameters of afebrile and white blood cell count are more established, the parameter of 'days till CRP ≦ 30% of maximum' is commonly used in Japan to assess the efficacy of antimicrobials towards infection [13]. Correlation tests were performed to investigate if there was any correlation between vancomycin susceptibility of the initial MRSA strains (AUC) of every patient with each of the above parameters. Results showed a significant positive linear correlation between AUC and the corresponding patients' initial therapeutic response parameters of 'days till afebrile' (*r *= 0.828, *P *< 0.01), and 'days till CRP ≦ 30% of maximum' (*r *= 0.627, *P *< 0.01). The less susceptible the MRSA strain was to vancomycin, the longer it took for the patient to become afebrile and for his/her CRP value to return to normal (Table 2). This supports Charles et al.'s results where patients infected by hetero-VISA took a longer time to defervescence [14], and also indicates that various levels of vancomycin susceptibility in MRSA strains play a critical role as a determinant in patients' clinical outcome in bloodstream infections. Therefore, it is desirable to identify such infected cases and to initiate optimal treatment for these infections early in the clinical course; good communication between the clinician and clinical laboratory is essential for this purpose. In addition, improvement in clinical outcome may be achieved if early consideration is given to patients who show a slow initial therapeutic response that could signify the involvement of MRSA strains with reduced susceptibility to vancomycin. Nevertheless, the initial therapeutic response parameter of 'days till WBC <10,000' could not be used as a determinant of patient's clinical course in our study. This is due to the reason that most of the patients enrolled in our study were either leukopenic or had leukocytosis even before the initiation of vancomycin, and therefore no conclusive data could be derived from this parameter.

In contrast to another report [14], the number of MRSA positive blood cultures could not be used as a parameter to assess the impact of MRSA vancomycin susceptibility on patients' clinical outcome in our study. Blood culture is not a routine test in Juntendo University Hospital, therefore there were no continuity of blood culture results for certain patients. In addition, we observed that MRSA blood culture positivity was not definitive of a patient's infection status. This could be seen in the case of patient no.15, as even though he had a MRSA negative blood culture after 4 days of vancomycin therapy, this patient's bloodstream infection worsened and VISA was isolated from his blood 3 weeks later, as opposed to the case of patient no. 13 where this patient had a relatively fast recovery from infection even though it took 18 days for his blood culture to be MRSA negative. Patient survival rate at one month was also not conclusive to predict the relationship between reduced MRSA vancomycin susceptibility and patient outcome in our study. This is because even though some mortalities in our study were due to MRSA bloodstream infection, there were also patients who died due to their underlying diseases such as cancer and heart failure.

Although our results were statistically significant, limitation of this study includes its small sample size and retrospective design. Patient sampling was difficult due to the strict inclusion and exclusion criteria employed in our study; for example, patients were frequently given vancomycin together with antibiotics of different classes. There were patients who died after one or two days vancomycin therapy due to the severity of their infection, patients who were not given vancomycin due to their underlying diseases, and there were also cases where vancomycin trough levels were not sufficient to meet the criteria. We did not include these cases into our current investigation as it might introduce bias into the study. An extended, progressive study would be needed to fully establish the correlation between patient clinical outcome and vancomycin susceptibility of the causative MRSA.

## Competing interests

The author(s) declare that they have no competing interests.

## Authors' contributions

HN carried out the laboratory investigations, participated in the design of the study and drafted the manuscript. SH participated in the design of the study and reviewed the medical records. MK helped in medical records review. TO provided strains for the study. FT contributed in statistical analysis. LC participated in the study design and reviewed the drafted manuscript. KH conceived of the study, participated in its design and coordination. All authors read and approved the final manuscript.
